# Market shaping for AT: scoping review and case study of an intervention

**DOI:** 10.3389/fresc.2026.1918680

**Published:** 2026-09-04

**Authors:** Ellie Cole, Victoria Austin

**Affiliations:** Global Disability Innovation Hub, University College London, London, United Kingdom

**Keywords:** assistive technology, case study, disability, market shaping, UNICEF

## Abstract

**Introduction:**

Assistive technology (AT) holds the potential to transform lives. With need projected to reach 3.5 billion by 2050 while access remains at only 10%, even basic AT is out-of-reach for most. Expanding access to AT is a complex systems challenge: AT markets are often small, underdeveloped and fragmented, particularly in low-income settings. Addressing market failures is essential to expand access, and market shaping has been introduced as a core tool since 2018.

**Methods:**

This paper presents a scoping review and a case study examining how market shaping for AT has progressed. The case study explores how UNICEF, one of the world's largest procurement actors, has conceptualised and implemented it. Fifteen publications were included in the scoping review and four themes emerged: systems-thinking; unintegrated innovation; governance and financing; and data gaps. The UNICEF case study draws on eight interviews, complemented by early procurement data provided by UNICEF on selected countries.

**Results:**

A recurring contestation was the need to move beyond siloed thinking to a coordinated and coherent systems perspective, recognising the multidimensional, interdependent factors influencing AT markets. To achieve this, several publications introduce “missions-thinking” as a complementary perspective. Evidence indicates that UNICEF's market shaping has had transformative impact to-date. Early data shows an average price reduction of 84% for hearing aids, and 34% for wheelchairs (the latter reduced by one county's outlier score of 5%).

**Discussion:**

Sector challenges persist, including funding and logistics, yet evidence suggests that oversight and leadership by a body such as UNICEF can support transparency to move towards fair and healthy AT. The paper concludes that market shaping for AT holds significant promise, but achieving its greatest impact requires coherent and bold stewardship, with market shaping as one tool within an “AT mission”. This has begun, and UNICEF's new strategic commitment to AT access shows promise for the future.

## 1 Introduction

Assistive technology (AT) holds the potential to transform lives, enabling people to live independently and participate fully in society. However, access to even the most basic AT remains out-of-reach for many. The scale of need is vast, with one-in-three people (around 2.5 billion globally) estimated to benefit from at least one form of AT (1). Demand for AT is increasing swifty, and the current need for AT is projected rise to over 3.5 billion people by 2050, due to an ageing global population, the rise in non-communicable disease, and chronic health conditions (1). This makes AT a critical issue for global development. However, despite the vital demand, access and availability remains deeply inequitable with over 90% of those needing AT not having access (ibid). People who need AT are disproportionately represented in low- and middle-income countries, where often only a tiny fraction have access to the devices they need (2).

Getting AT to the people who need it most is a complex systems challenge. AT markets are often small, underdeveloped and fragmented, particularly in low-income settings (3) or where products are highly specialised (4). Limited or inconsistent demand results in weak incentives for suppliers to enter markets or innovate products, fragmented supply chains and uneven availability (5). Consequently, in many areas, availability AT is often limited, of inconsistent quality, or only at prohibitively high cost (2). Understanding and addressing these market failures is therefore essential to expanding access to lifechanging AT at scale.

This paper sets out to address the central question of how market shaping in the AT sector has progressed since it began in 2018, with a focus on the role of public sector intervention via the UNICEF supply catalogue. It is divided into five sections. After introducing key concepts, section two presents a scoping review of the recent market shaping for AT literature. Section three presents a case study of UNICEF's market shaping activities and results from a series of key informant interviews and procurement data from the UNICEF supply catalogue. The themes emerging from the scoping review are used as an analytical frame to interpret these results. These findings are discussed in section four, and finally section five presents the conclusion.

### 1.1 What is AT?

Assistive technology is an umbrella term which encompasses both products designed to enhance functional ability (such as wheelchairs, hearing aids, eyeglasses and communication devices) and associated services, which include assessment, rehabilitation, user training, and ongoing device maintenance. To improve access to assistive products, WHO (6) introduced a “priority assistive products” list of 50 items selected based on population need and potential impact. This list is intended to support countries to meet their commitments under the Convention on the Rights of Persons with Disabilities (7) and the Sustainable Development Goals (SDGs, 8).

Beyond improving functional ability, AT has wider social and economic implications. It plays a central role in enabling people with disabilities or with functional difference to access education, employment opportunities, and community life (1). AT can be lifechanging, expanding horizons of independence and participation, enhancing wellbeing and enabling individuals to do what matters most to them.

### 1.2 Market shaping for AT

Market shaping for AT was established at the 2018 Global Disability Summit in London as a core mechanism for reducing the price of assistive products. The summit also saw the establishment of the Global Partnership on AT, ATscale, set up for this purpose (9). Market shaping is a tool that has been used in other development initiatives in global health and refers to a suite of coordinated actions that are designed to address asymmetry, imbalance, gaps and bottlenecks and enable healthy markets for products (10). The aims of market shaping interventions are therefore to ensure that quality products are available and affordable where they are needed, while simultaneously providing sufficient incentives for actors to remain sustainably engaged in markets and to innovate products (11, 12). Although there is no single definition of “market shaping”, and the approach taken varies between actors, activities typically aim to tackle one or more shortcomings in the “5As”: affordability; availability; assured quality; appropriate design; and awareness (10). Market shaping approaches have been used extensively in the vaccine space (13–15), as well as goods such as insecticide-treated mosquito nets (16, 17), and HIV self-tests and antiretrovirals (18).

Increasingly, market shaping is not considered a “magic bullet” but as one element working alongside policy reforms, programmatic interventions, and innovation to drive positive and sustainable change in product and service delivery (19). Market shaping initiatives have been seen as very successful in some cases, for example in reducing the price of first-line antiretroviral medication by up to 99% (20). But can be difficult to assign a specific causal relationship between price reduction and market shaping activities (18), as these initiatives often interact with dynamic networks of interconnected actors and factors that are both interdisciplinary and interdependent (21, 22). Due to the nature of AT, which often requires services as well as provision (23), market shaping for AT must also account for this complexity, as any breakdown in the complex supply chain can prevent “life-saving and life-enabling” AT from reaching those in urgent need (24).

In many low-income settings, markets for AT and the services that surround them, are small, fragmented or in some cases may be entirely absent. This requires the intervention of “market making” activities, which are often a necessary precursor to market shaping. Market making involves stimulating demand through creating awareness of available products and their benefits and establishing supply infrastructure (23). ATscale aims to foster exactly this “enabling environment” for AT to scale through supporting policy reform, system strengthening and mobilising investment (25).

It is therefore important to understand the current situation of market shaping for AT. In 2018, MacLachlan and colleagues (5) published a scoping review on market shaping as background for the development of the SMART matrix (systems-market for assistive and related technologies), which is the precursor to the present scoping review. Market shaping for AT is graining traction, but the field has changed substantially over the last decade since ATscale was launched. Its mandate is to reach half a billion more people with lifechanging AT by 2030, with a core pillar of its strategy being developing and energising AT markets. UNICEF, one of the most influential market shaping actors, is a founding partner of ATscale. The FCDO-funded AT2030 programme, also established in 2018, invested in UNICEF to conduct the global procurement necessary to add several assistive product categories to the supply catalogue it shares with WHO, to aid the pooled procurement mechanism of market shaping. The catalogue stimulates supply and demand, and uses large-scale procurement to stabilise and expand markets, but is operated from a public sector perspective (11), differently from some traditional market shaping efforts which primarily work with private sector actors. Building on this momentum, in 2025 WHO launched a guide specifically focused on AT market shaping and analysis (23). The guide offers a six-point approach to market shaping which aligns with the 5P model. This model comprises five interconnected areas needed to ensure equitable access to AT: people; policy; products; provision; and personnel (1).

Market shaping for AT is a rapidly evolving field. Therefore, identifying how these strategies are operationalised is essential to strengthen AT systems and promote availability and access. The present paper presents a scoping review and case study to explore how market shaping has been conceptualised and implemented by one of the world's largest public sector actors. To do this, the research seeks to answer two research questions:

1. How has market shaping for AT evolved since it started in 2018?
2. What impact have UNICEF's market shaping for AT strategies had to-date, and what conditions are needed for it to achieve its fullest potential?

## 2 Scoping review

### 2.1 Objective and rationale

The aim of this review was to identify publications about market shaping that have been published since 2018, to build upon a previous scoping review on the subject (21). Scoping reviews are an effective way of synthesising literature where evidence is heterogeneous, methodologically diverse, or where research and data are emerging (26). This review follows the scoping review extension to the widely used PRISMA guidelines (27), which recommends completing a checklist to promote rigour (see Supplementary File One for the completed checklist). No protocol was published for this review. However, all refinements to search and analysis strategies were documented to support transparency.

### 2.2 Methods

#### 2.2.1 Inclusion criteria

▪ In English
▪ Journal publication
▪ Central focus on market shaping for AT
▪ Published from 2018 (inclusive)

#### 2.2.2 Exclusion criteria

▪ Not meeting the above criteria
▪ Full-texts being unavailable
▪ The precursor review itself (21)

#### 2.2.3 Information sources

To update the MacLachlan et al. scoping review (21), a search was conducted on the same databases and journals (Table 1) with the University College Dublin database (Stella Search) being replaced by the comparable University College London database (UCL Explore). To maintain consistency with the precursor scoping review and replicating its approach, the search was also restricted to journal publications. Key grey literature, such as market shaping primers and frameworks (10, 28, 29), and AT specific market shaping literature (23, 30, 31), was used to support the results synthesis.

**Table 1:** Scoping review information sources.

Database	Journal
Taylor and Francis Online	Disability and Rehabilitation: Assistive Technology
ProQuest	Assistive Technology Journal
Science Direct	Technology and Disability Journal
PubMed	Journal of Enabling Technologies
UCL Explore	Journal of Assistive, Rehabilitative and Therapeutic Technologies
SCOPUS	
EMBASE	
SpringerLink	
National Library of Medicine	
WHOLIS	
JSTOR	
Emerald Insight	
ASSIA	
REHABDATA	
LILACS	
CIRRIE	
SCIE	
AIM (African Index Medicus)	

#### 2.2.4 Search

The review employed a flexible search strategy, with terms including “market shaping”, “market making”, and “assistive technology*”, “assistive product*”, “assistive device*”. The search strategy was developed through discussions by the authors, and the date of the last search was 28th April 2026. Supplementary File Two contains the strategy and search string for the PubMed database as an example.

#### 2.2.5 Selection of sources

The preliminary set of inclusion criteria were developed in discussion between the authors, and are aimed at updating the previous scoping review (21). A form was developed with fields such as publication type, conceptual focus and the kinds of insights the publications provided (empirical, conceptual or policy). The first author conducted the screening with support from the second author in cases of discrepancy or uncertainty. To ensure consistency, inclusion and exclusion criteria were developed by both team members based on those used in the precursor review, Regular discussions were held to review selection decisions and resolve any disagreements. No third-party adjudicator was required in the screening process.

The results of the searches were imported into Zotero (version 9), and publications were cleaned for duplicates and screened for basic ineligibility. A two-stage screening process was then conducted where potential publications were first assessed on title/abstract and then through a full-text review. Through this process 15 publications were included in the review.

#### 2.2.6 Data charting

Information was extracted from the collated publications and summarised using a template developed for this review. The extraction form presents publication details, study characteristics, and key market shaping themes and findings. The data charting form was updated iteratively throughout the review process.

#### 2.2.7 Data items

Data were abstracted based on bibliographic details, article characteristics, market shaping frameworks and actors, and key findings.

#### 2.2.8 Synthesis of results

**Figure 1:**
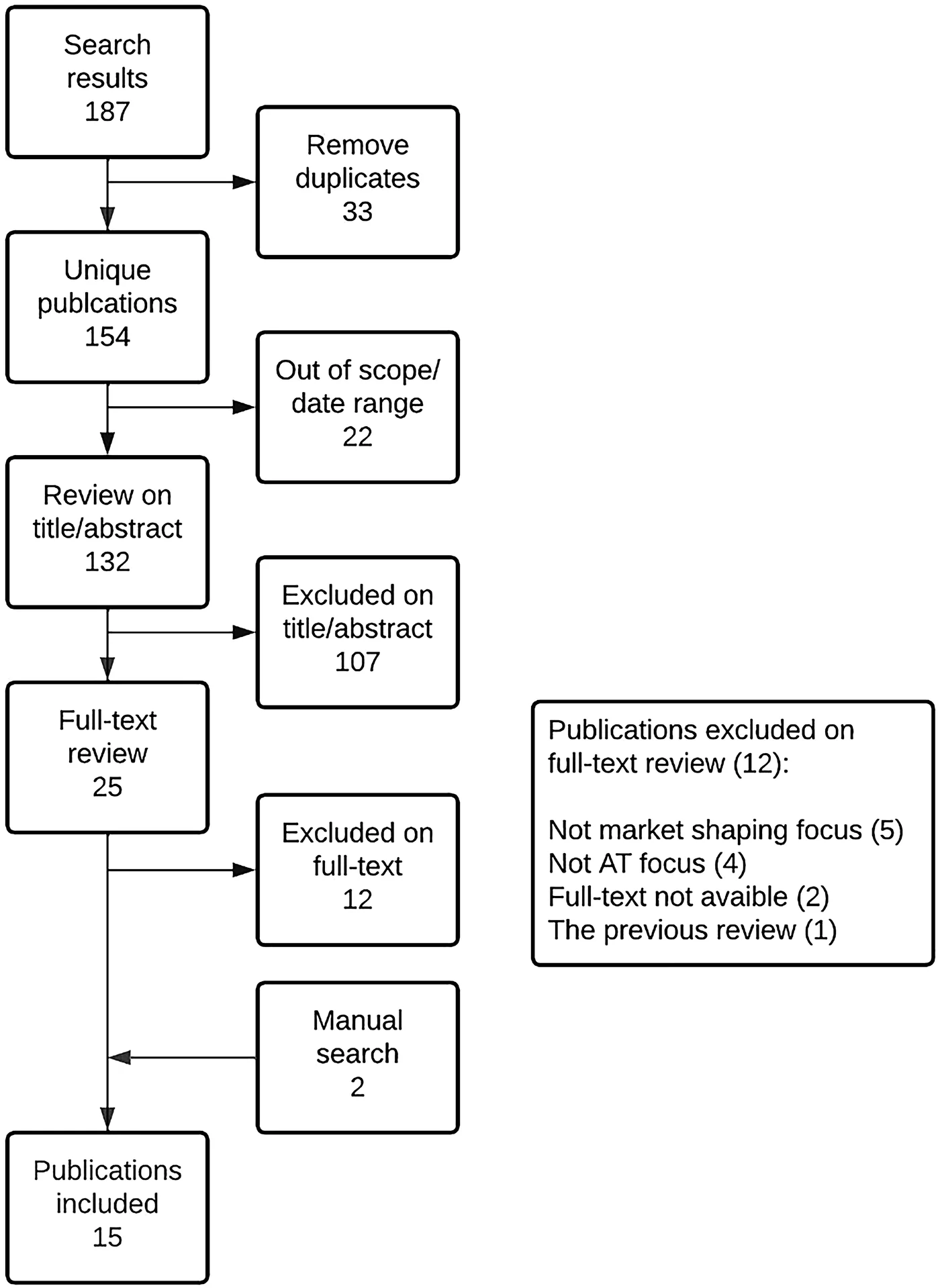
Scoping review selection of sources of evidence flowchart.

Results from database searches are presented in a flowchart (Figure 1) and information from the included publications is summarised and presented in a table (Table 2). The synthesis of these searches is presented as a narrative.

### 2.3 Results

A total of 187 publications were identified through the searches. After these were cleaned to remove duplicates, those that were out of the date range or scope of the review, the remaining 132 were assessed on title and abstract, of which 107 were excluded. The full text of the remaining 25 publications was reviewed, with a further 12 being excluded. Two publications were added following a manual search, leading to a final total of 15 publications.

#### 2.3.1 Characteristics

In this review, a diverse set of evidence types emerged. These included commentaries and position papers, case studies, literature and scoping reviews, policy and economic analyses and product narratives. The focus of publications was also broad, including market shaping paradigms (5, 21, 24, 32–34), structural and market failures, including weak supply and demand, limited competition, and fragile supply chains (3, 4, 24, 32), and disruptive innovation models (34). Publications were frequently responses to international AT summits (5, 35, 36) and data (or lack thereof, 34, 36, 37). Few publications focused on types of assistive devices, with only one publication each about wheelchairs (3) and prosthetics (38). The publications span 2018 to 2026.

#### 2.3.2 Results of sources

Table 2: characteristics of sources of evidence.

**Table 2:** Characteristics of sources of evidence.

#	Publication	Type	Key themes
1	Danemayer et al. (37) Measuring assistive technology supply and demand: a scoping review	Scoping review	▪ Market shaping requires both supply and demand data ▪ Lack of harmonised AP definitions and indicators undermines population data ▪ Evidence heavily skewed to vision/hearing products (76%), USA overrepresented ▪ Supply-side data extremely lacking
2	Holloway et al. (34) A review of innovation strategies and processes to improve access to AT: looking ahead to open innovation ecosystems	Literature review and case studies	▪ Product-centric innovation dominates AT literature (76%), while case studies predominantly focused on supply ▪ Most innovations in products, but in provision and supply less well-documented. ▪ 2 high-level themes: open innovation; disruptive and radical innovation. ▪ Disruptive APs often accelerated by digital advances ▪ Absence of standards and data constrains demand creation ▪ “open innovation” could diversify markets and lower entry barriers for smaller actors
3	Layton et al. (32) Assistive technology as a pillar of universal health coverage: qualitative analysis of stakeholder responses to the world health assembly resolution on assistive technology	Content analysis	▪ Systems approach critical for AT to be an effective pillar of UHC—both an end and a means ▪ Weak market competition, tariffs and poor quality control restrict access ▪ AT must be embedded in broader health-system strengthening to shape equitable markets ▪ Systemic coordination across sectors needed to correct market failures to provide access to quality and affordable AT
4	Layton et al. 2020 Opening the GATE: systems thinking from the global assistive technology alliance	Position paper	▪ Regional multi-stakeholder alliances have formed as fora to foster access to quality and affordable products, research into usability, quality and abandonment ▪ Influence markets through the 5Ps from national to global levels ▪ Global Alliance of Assistive Technology Organisations (GAATO) acts as a facilitator of action
5	Layton et al. (33) Towards coherence across global initiatives in assistive technology	Position paper	▪ AT landscape is expanding but fragmented ▪ GATE should operate across agencies and sectors, including working closely with GAATO ▪ Pooled donor funds can enable GATE to allocate funding led by the global research agenda
6	MacLachlan (39) Access to assistive technology, systems thinking, and market shaping: a response to Durocher et al.	Comment	▪ Argues that ‘fairness of access’ to AT is not sufficient and systems thinking and market shaping approaches should be adopted ▪ Deliberative systems-thinking in AT is needed to overcome ‘funnel of inaccessibility’
7	MacLachlan et al. (35) Assistive technology policy: a position paper from the first global research, innovation, and education on assistive technology (GREAT) summit	Position paper	▪ APs are both mediators and moderators of social change ▪ AT policies should promote 5As (+AA—affordability and acceptability) ▪ Highlights the importance of policy in market shaping ▪ Unified national AT systems can aggregate demand to stabilise markets
8	MacLachlan and Scherer (5) Systems thinking for assistive technology: a commentary on the GREAT summit	Commentary	▪ Examples of systems/non-systems thinking across 10Ps shapes markets ▪ 5Ps + procurement, place, pace, promotion and partnership ▪ Emphasises user-centred, context-sensitive approaches are needed to avoid supply-driven, inequitable market outcomes ▪ Argues that systems/non-systems thinking are at ends of a continuum
9	Onley and Dickinson (4) Australia's new National Disability Insurance Scheme (NDIS): implications for policy and practice	Policy analysis/qualitative research	▪ Aims to address inefficiencies in the previous service-based system ▪ Rapid transition to individualised funding created immature AT markets ▪ Government ‘layered stewardship’ of emerging markets is needed to ensure equitable access ▪ Demand constrained by lack of knowledge of entitlements ▪ Highlights how intersectional factors constrain ‘choice and control’
10	Pfuhl and Reinecke (38) Ottobock as a case for globalization of medical devices	Case study	▪ Ottobock shapes global prosthetics markets through controlling the vertical ‘value chain’, proprietary clinics and standards-setting ▪ Frames international expansion as core to growth and innovation ▪ Argues premium strategies can influence and ‘uplift’ market standards in emerging economies ▪ Positions itself as a ‘responsible corporate citizen’ while consolidating market power
11	Savage et al. (24) Applying market shaping approaches to increase access to assistive technology in low- and middle-income countries	Product narrative synthesis	▪ Nascent AT markets in all low- and middle-income countries ▪ Root causes of market shortcomings: weak enabling environment, insufficient market information, high transaction costs, risk imbalances ▪ Market shaping interventions should include reduce transaction costs, increase market information, balancing risk, innovative financing mechanisms ▪ WHO APL list can encourage investment/uptake ▪ Mission-thinking can prioritise wider social and economic value of AT
12	Savage et al. (3) Applying market shaping approaches to increase access to assistive technology: summary of the wheelchair product narrative	Product narrative/illustrative case study	▪ Wheelchair markets highly fragmented and dominated by low quality products and a huge number of manufacturers ▪ High shipping costs, import duties, and small volumes (limiting purchasing power) raise prices
13	Smith et al. (36) Assistive technology products: a position paper from the first global research, innovation, and education on assistive technology (GREAT) summit	Position paper	▪ Describe needs, opportunities and issues in the AT lifecycle ▪ AT lifecycle is complex, from design- to service delivery states, requiring multistakeholder coordination to shape markets ▪ Empasis on context-sensitive user-centred design and implementation—no ‘one-size-fits-all’ ▪ Suggests for disruptive AT, sometimes ‘push’ (supply) needed where ‘pull’ (demand) is absent. ▪ Data are needed for evidence-based product development
14	Vallès Codina (42) The purely economic case for investing in assistive technology and health in lower and upper-middle income economies: input-output and network analysis of the assistive technology and health industrial ecosystem.	Input-output and network analysis	▪ Investment in AT/health sectors produces immediate economic gains ▪ Triggers ripple effects—AT/health sectors highly connected in national production systems ▪ AT investment generates strong upstream spillovers across 75%–99% of industries in national economies; ▪ Argues AT as a strategic sector for industrial policy and market shaping ▪ Cost-benefit-analysis can miss these dynamic effects
15	Tay-Teo, Bell and Jowett (22) Financing options for the provision of assistive products	Literature review and interviews	▪ AT chronically deprioritised and underfunded due competing government priorities ▪ Small market size causes perceived investment risk, limiting growth, ▪ Identifies seven financing mechanisms to expand and stabilise markets ▪ Argues for improved and efficient governance to align financing, procurement and provision

### 2.4 Synthesis of results

This scoping review synthesised the literature around market shaping published since 2018 which fell into three broad categories: publications reporting primary data analysis; review papers; and comments and position papers, which predominantly followed the Global Research, Innovation, and Education on Assistive Technology (GREAT) summit. Several thematic areas emerged comprising: systems-thinking framing; active but unintegrated innovation; governance and financing challenges; and data gaps. We now discuss these in turn.

#### 2.4.1 Systems-thinking framing: market shaping or mission-led?

The need for systems-thinking is consistently raised across the literature. This approach, some authors argue, is needed for AT to be equitably allocated “across the population and life course” (34). Authors drew on a range of frameworks to assess market shaping, including the SMART matrix, originally developed in the precursor review (21). Using this framework, Holloway et al. (34) found that while there was good coverage of AT innovation across most matrix domains, there was a lack of evidence about AT in optimal markets at national or population-levels. This gap is interpreted as both the lack of data, but also as an indication that optimally functioning markets are largely absent.

Systems-thinking regards markets as part of a “complex adaptive system”, which is underpinned by the organised knowledge, procedures and policies that shape how AT is used and evaluated (5). Whereas market shaping approaches acknowledge the critical role of multi-stakeholder, cross-sectoral partnerships, systems-thinking is more multi-disciplinary and may include partnerships with actors such as academia, NGOs (often as delivery partners able to access communities and last-mile populations), as well as civil society and AT users themselves (24). This shift recognises the multidimensional and interdependent factors that influence market shaping, which cannot be addressed in isolation. While authors recognise that approaches will not apply uniformly in all contexts (or at all times even within the same context), systems-approaches can bring diverse elements into view (5). Acknowledging this, MacLachlan and Scherer propose expanding the 5Ps to 10Ps to include procurement, place, pace, promotion and partnership (5), areas of key concern in effective market shaping.

Several publications introduce “missions-thinking” as a complementary perspective to market shaping (5, 24, 34, 39). This perspective suggests that a comprehensive and sustainable approach to universal AT access requires understanding it as a public policy “mission”. An “AT access mission” considers the cross-cutting socioeconomic challenges and transformations, as well as a it being a technical activity (24). By mobilising actors across sectors and focusing resources on a shared social goal, an “AT access mission” would be ambitious, but has the potential to create transformative and sustainable change.

Layton et al. (32) argue that AT should be a central pillar of universal health coverage (UHC). They highlight the need for effective international cooperation, and that through market shaping ATscale (40) can “align and consolidate” these efforts. In a related publication Layton and colleagues (33) further call for greater coherence across the global initiatives in the AT landscape, proposing that a “multi-donor trust fund” could be established. Such a mechanism could pool resources, aggregate demand, and steer investments to promote a more sustainable global AT landscape. This has been attempted by various actors but the current economic situation as well as changing geopolitical landscape means that to-date it has had limited success.

Savage and colleagues (3, 24) argue that recurring market failure issues are the same at both local and global levels. In their wheelchair market analysis, Savage reported extreme market fragmentation, which was driven by a proliferation of suppliers, and a predominance of poor-quality wheelchairs in low-income settings. The author also reports that where markets do exist in such settings, they focus primarily on wealthier segments of society, predominantly in urban areas, leaving those in rural and remote areas underserved or only able to access low- and often inappropriate devices (24).

#### 2.4.2 Innovation needs integration

This review found that innovations were not always easily or effectively integrated into market shaping. Some authors argue that AT is often still framed as a single solution to a discrete problem, and the focus remains on the product, rather than the process, which may not necessarily reflect the needs of AT users (5). In such cases, innovations of the system are often seen as independent of innovations of the product.

Smith et al. (36) suggest that sometimes a “push” from the supply market is needed in cases where the demand (“pull”) is absent. From the review the Ottobock corporation is an example supplier-led “push” innovation (38). The organisation introduces “premium” prostheses into under-developed settings and build up the demand over time, eventually causing—they argue—an “uplift” to the whole market. The corporation has also introduced innovations in its supply chain through the establishment of proprietary clinics for the distribution of its patented products and a cloud-based management system aiming to develop an “interconnected healthcare ecosystem” (38).

Several studies highlighted digital products as an area for future innovation (34, 36, 37). Digital AT is an umbrella category encompassing devices ranging from AT-specific digital products such as screen readers or eye-gaze systems through to smartphone apps. Because of this, digital innovation is challenging to measure as there are inconsistent definitions and functional domain crossover (37). Both Holloway et al. (34) and Smith et al. (36) discuss “disruptive” innovation. The authors argue that this kind of innovation could be an opportunity for companies to invest in new technologies or novel strategies to enter unhealthy markets and improve supply chains. Predominantly driven by digital advances, disruptive elements also included local production (such as 3D printing) and point-to-point distribution (such as Amazon).

#### 2.4.3 Governance, financing challenges, and “what counts”

The case for investing in AT is strong. Existing evidence points to a 9:1 return on investment in AT (41), showing the scale of the missed opportunity. Complementing this, in an input-output and network analysis, Vallès Codina (42) found that investing in AT value chains has a positive spillover effect in 75%–99% of industries throughout the entire economy. This suggests that AT industries are in integral to most other sectors in the industrial ecosystem.

Issues of financing and governance are reported as key obstacles to effective market shaping for AT. MacLachlan and Scherer (5) state that often when AT is mentioned in policies, it is often only as a subsection, not as a coherent cross-sectoral policy vision, and with a lack of budgetary allocation. Few publications offered or potential solutions or data at a structural level. An exception is Tay-Teo et al. (22) who set out a number of different approaches to financing AT, ranging from taxation and social security down to crowdfunding. Each model had inherent strengths and weaknesses, depending on type of AT, the maturity of national systems and socioeconomic contexts. An example of this was discussed in Olney and Dickenson (4) who argued that the ambitious rollout of Australia's National Disability Insurance scheme resulted in rushed market shaping processes. They highlight the risks of rapid, large-scale policy reform in contexts where AT markets are severely under-developed and where governance architecture is not in place to steer such a significant structural change. To address these weaknesses, the authors call for “layered stewardship” to clarify governance, strengthen coordination to actively shape markets to promote universal equitable access to affordable AT (4).

Standards-setting is a central mechanism of market shaping and a means of exerting power. MacLachlan and Scherer (5) argue that global standards often function as “absolutes”, developed by industry actors in resource-rich countries or by multinational corporation, which may not reflect the situation in low-resource settings. For example, as the largest manufacturer of “premium” prosthetics, Ottobock can set technical standards that align with its own products and business model (38). Related to this, Savage et al. (3) argue that ISO minimum standards do not test for the environmental and contextual factors that are present in many low-resource settings, such as rough terrain, high humidity or frequent exposure to water. Due to these factors, authors propose a more relativist approach to standards (3, 5, 36). However, as standards-setting is one of the key principles of market shaping, the enthusiasm to make such a change may be minimal, and what the end result of this sort of transformation would look like is unclear.

Understanding the impact of market shaping then also requires an understanding of the perspective from which one observes. There is also a question of market shaping for whose benefit. In a case study of the Ottobock corporation, Pfuhl and Reinecke (38) demonstrate how a for-profit multinational organisation can shape markets by controlling the entire vertical “value chain” from design and distribution, down to product provision at proprietary “clinics”. While the company presents itself as a benign and “responsible corporate citizen”, its market shaping activities are focused on maximising its own growth and profitability, rather than getting quality and affordable AT to the people who need it the most.

It is well documented that systemic coordination is needed to ensure effective AT access (32). If a multistakeholder approach is needed where government proactively and transparently collaborates with donors and with civil society, it is logical that market shaping initiatives are also necessary. Good governance with the strong input from AT users can create the conditions for effective systems strengthening, where market shaping supports reliable delivery promoted by the creation of regular demand (5).

#### 2.4.4 Data gaps

As the AT sector expands rapidly, robust empirical data are needed for market shaping to be equitable for all. However, the review revealed extensive and persistent gaps in data, which undermines effective intervention (34, 37). Market shaping requires both robust demand and supply data. However, supply-side data are exceptionally limited (37). Holloway and colleagues (34) argue that such lack of data and standards undermines demand creation for AT, limiting the ability of smaller firms and innovators to enter and diversify the market.

In their scoping review, Danemayer et al. (37) found that a lack of common AT definitions and indicators undermined the collection of population-level data, and that existing data were dominated by hearing and visual AT (76% of all studies). These distortions can lead to significant impacts on policymaking and financial decision-making. MacLaughlan and Scherer (5) argue that procurement decisions that are based on this kind of skewed population estimates, or where they are disproportionately driven by demands of a vocal minority, can reinforce existing fragmentation and disrupt coordinated market shaping efforts. Without reliable data, market shaping risks reinforcing systems of exclusion, particularly around equity of affordable access for AT users in rural and remote areas, and for users with complex or fluctuating needs (4).

We now use these themes to consider the data from the UNICEF case study.

## 3 UNICEF case study: market shaping data and interviews

To complement the scoping review a case study of UNICEF market shaping for AT was developed.

### 3.1 Methods

Eight individuals participated in interviews, who comprised three people working on procurement, two AT technical staff and three people at country- and regional-level across UNICEF and partner organisations. The information from these participants was synthesised to form a case study of UNICEF's market shaping activities. Supply catalogue pricing data provided by UNICEF were also included where available. The emergent themes from the scoping review were used as an analytical lens through which to consider this case study data which is presented accordingly below.

Individuals were eligible to participate if their role included knowledge of UNICEF's global work on AT, including procurement and programmes. A purposive sampling strategy was used with individuals being invited to participate by the authors. The information sheet and consent form made it clear that participation was voluntary, confidential and that their views were their own. Characteristics of roles have not been listed to avoid breaching confidentiality, but participants included administrators, buyers and stakeholders of UNICEF AT procurement.

Data collection took place remotely over Teams between late 2024 and early 2026 using a topic guide specifically developed for the research. Interviews were transcribed using Teams' auto-transcription mechanism and checked for accuracy. These were not reviewed by participants, but were available on request. Data were analysed using inductive thematic analysis with manual coding being undertaken by one author with regular reviews to ensure consistency.

Interviews were conducted by the authors, who have extensive expertise in assistive technology and disability. Both authors are based at GDI Hub, and given their proximity to the AT2030 programme and UNICEF, care was taken to manage this positionality. This involved systematic cross-checking of ongoing analysis to identify assumptions and regular team discussions to review analytic choices throughout the research.

### 3.2 UNICEF and market shaping

UNICEF has become a central actor in the market shaping space. The organisation—as one of the largest procurers of global products, such as vaccines for low-income nations—has a “catalytic” role in shaping markets. Its approaches include market analysis, reducing supplier uncertainty and lowering prices by aggregating demand, setting quality standards, and vetting and negotiating long-term agreements with suppliers (43). UNICEF's Supply Division works through partnerships and strategic procurement to reduce market barriers, improve prices and secure a stable supply of essential products for children in low- and middle-income countries. The UNICEF/WHO supply catalogue is one of the world's largest procurement platforms, listing more than two thousand pre-vetted items.

The supply catalogue is available to selected actors, including governments, other UN agencies, registered NGOs and philanthropic organisations. Products in the catalogue are listed following competitive bidding with suppliers that have completed a pre-vetting quality-assurance process. Indicative pricing is set per unit, which does not include estimates for additional fees, such as shipping, handling, or import duties (if exemptions are not in-place). The lead-in time from procurement to delivery is typically to be around six-weeks. In 2025, UNICEF procured $5.6 billion in supplies and services in 164 countries (44).

**Figure 2:**
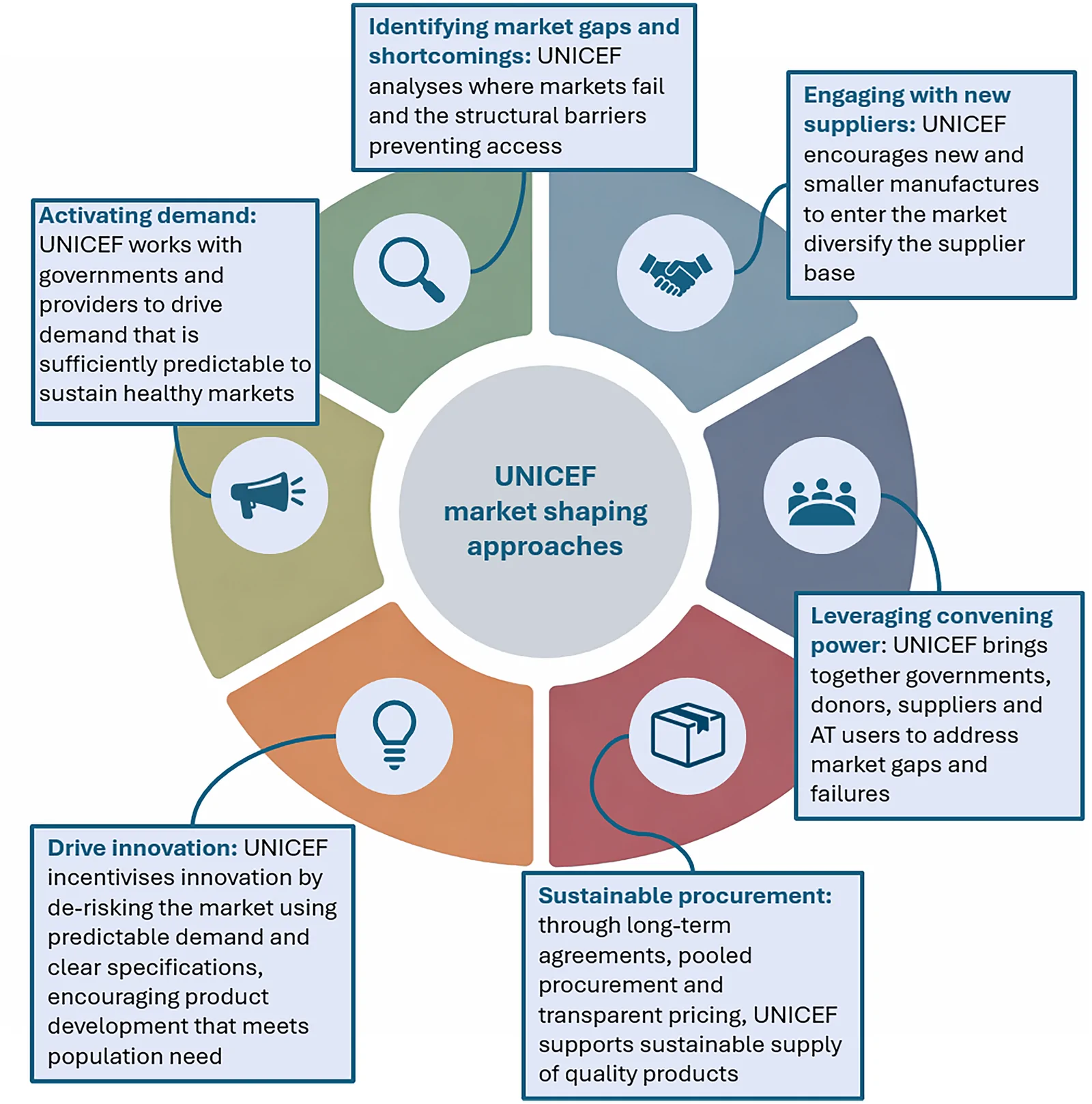
UNICEF market shaping approaches—design by author based on UNICEF supply strategy (50).

UNICEF's market shaping approach is made up of six core actions (Figure 2). These comprise activating and stimulating demand; identifying market gaps and shortcomings; engaging with and encouraging new suppliers to enter markets; leveraging its convening power to bring together stakeholders and foster collaboration; procuring supplies at-scale at sustainable prices; and incentivising innovation and improving solutions.

In response to the huge and growing unmet need for AT, in the last half decade UNICEF began to add assistive products to the supply catalogue via Global Procurement Tenders in AT product categories. UNICEF have now done the procurement to add more than 200 products across six categories: mobility, vision, hearing, selfcare, digital AT and inclusive education (44). The catalogue prices relate to the assistive products themselves and do not include the broader costs of assistive technology provision, such as assessment, fitting, user training, follow-up and maintenance. However, the addition of these products the supply catalogue marks a notable shift in how AT is positioned within global supply systems, which is partly the result of strong and coordinated work across the AT sector by global partners including WHO, UNICEF, ATscale and the AT2030 programme. AT access was supported by significant political backing, including the Global AT Resolution (WHA71.8) adopted at the 71st World Health Assembly in 2018, which was followed by the Global Report on Assistive Technology (1).

UNICEF and the WHO's assistive products specifications were developed through AT2030 in 2018–2020 (45). Together with the subsequent UNICEF/WHO public procurement manual (45) they provide the basis for normative quality standard for suppliers. The inclusion of AT in the UNICEF catalogue aims to ensure that these products—such as wheelchairs, eyeglasses, hearing aids and communication devices—are available as essential products at a reasonable price and quality. One particular benefit is that the products become available to resource-constrained nations as UNICEF has already negotiated these (better) terms at a global level. Applying UNICEF's market shaping expertise to the AT sector has the potential for innovation and great impact in a sector that has often been characterised by inconsistent and low levels of demand, market fragmentation, and weak quality assurance.

The data used for this paper were provided by UNICEF Supply Division from its initial procurement records Although these data are currently limited as many products are new to the catalogue, they nonetheless provide valuable insights into early trends that have not been reported elsewhere. Despite the limited available data, early evidence reveals that UNICEF's market shaping strategies have already shown significant success.

**Figure 3:**
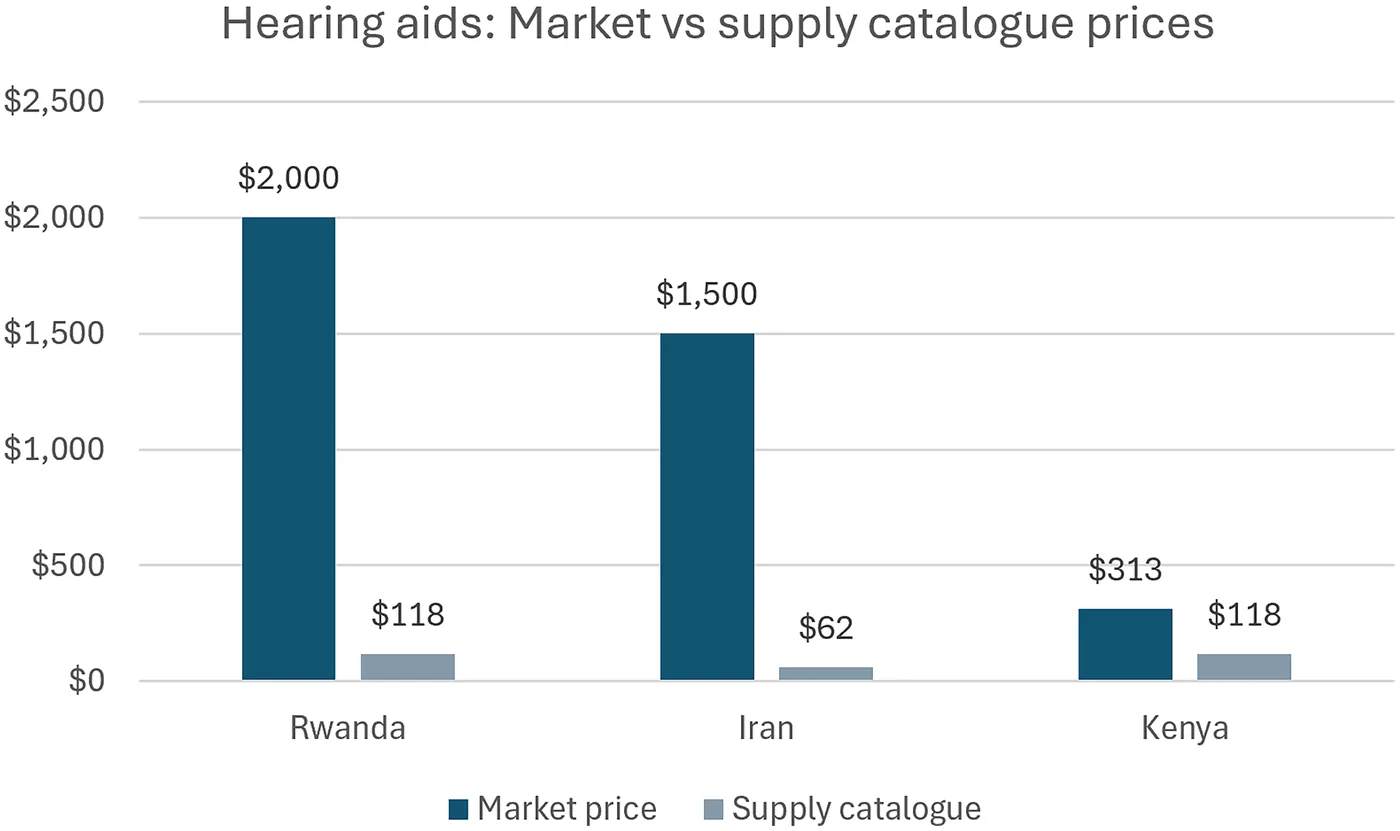
UNICEF pricing data: hearing aids market verses UNICEF catalogue negotiated price.

Hearing aids are the product for which we have the most data (Figure 3). In Rwanda the cost of hearing aids plummeted by 94% from $2,000 per device to $118 (46). In Iran, a 96% reduction in the price of hearing aids was noted, from $1,500 to $62. In Kenya the price drop was 62% ($313 to$118). Although data are only just beginning to emerge for other countries, the scale of this evidence reveals a significant impact. The average drop by country is 84% (notwithstanding that purchase frequency differs). This evidences the huge potential of market shaping for AT, particularly in places where there is limited local production, or a limited knowledge base or capacity to negotiate better supply deals. This should not be underestimated at a time of resource constriction.

**Figure 4:**
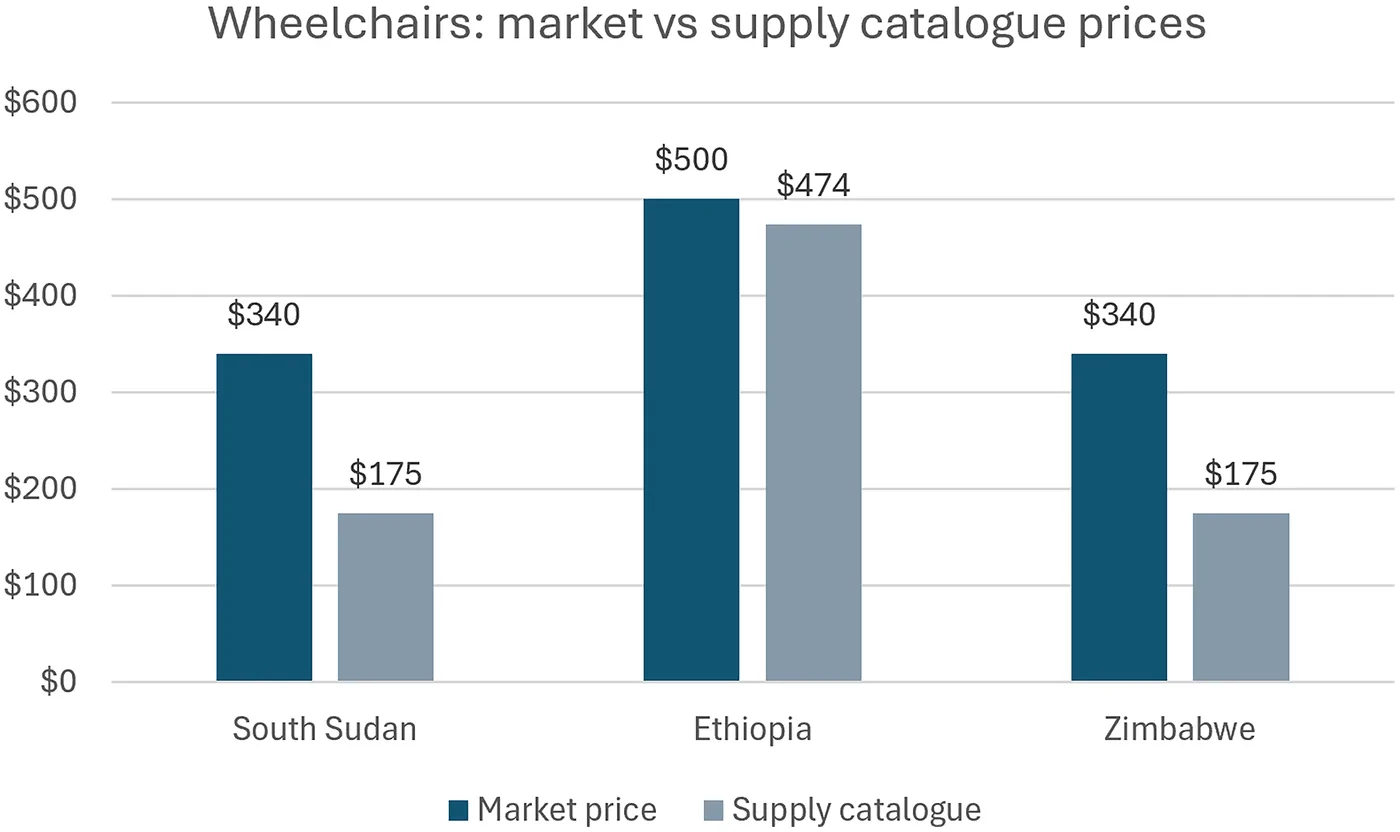
UNICEF pricing data: wheelchairs, market verses UNICEF supply catalogue negotiated price.

In the case of wheelchairs—the other product for which we have data—prices have shown a comparatively more moderate reduction thus far. In Ethiopia (Figure 4), the change in price was just 5% (from $500 to $474). This kind of moderate reduction may be due to a greater number of suppliers already operating in that location; more developed local production; or an already well-serviced wheelchair market. In both Zimbabwe and South Sudan, the reduction in price for wheelchairs was the same 49% (from $340 to $175). This gives and average price reduction, by country across the three countries of 34% (notwithstanding the purchase frequency differs).

These are only illustrative early findings. Limited pricing data are available at present, and we are therefore unable at this stage to model for exclusion of potential outliers (e.g., wheelchairs in Ethiopia which might have specific contextual conditions). Nonetheless, the data already show that the markets for wheelchairs and hearing aids do vary both by product, as well as country context, and we believe the price reductions to be linked strongly to UNICEF's market shaping activities.

### 3.3 Results

#### 3.3.1 Systems level thinking

The data show that AT is consistently framed as a systems problem, which is helpful and aligns with the scoping data. While respondents were in broad agreement that market shaping for AT can be an effective tool, there was less consensus about where activities are most effective within market systems. Some respondents highlight that market shaping should stem from global demand creation, but others argued that that for them market shaping must start from the grassroots, with AT users' needs and contexts, and finds ways to simultaneously support local manufacturers and supply ecosystems. One respondent went further, arguing that bottom-up market shaping was the only way it could be done ethically, avoiding the reproduction of colonial or paternalistic power dynamics. UNICEF do in practice encourage local supply and procurement where it is available and of a good price and sufficiently high quality.

The inclusion of AT in the supply catalogue is intended to plug a gap where local procurement isn't available. Nonetheless, the point about the engagement and involvement of users and local suppliers, remains key. This is the reason that partners like ATscale have deliberately involved smaller suppliers and AT users in their partnerships. Participants also emphasised that the structural and systemic gaps in the AT markets cannot be solved through market shaping alone, which also goes to this point, arguing that market shaping should not be seen as ‘silver bullet’ and that strategic investments are needed across the whole AT ecosystem. UNICEF concur, and its new global strategy (2026–2029) centres AT as one of only a few organisational targets around which to build partnerships and address resources (47).

Some participants argued that the complexities of AT markets were a challenge to effective market shaping. The infrastructure around AT is much more complex than that of other non-food items (NFIs), where “it's distribution, not provision”. Unlike most NFIs, the risk of adverse outcomes is much higher, as when AT is not properly assessed and fitted it can even lead to harm, and this is well understood, with one participant giving an example of poorly fitting devices leading pressure sores, complications from which can be fatal, a situation where, they argued, “rehab becomes wound care”.

For many, demand-side market shaping is critical. UNICEF undertakes activities of “demand creation”, aiming to build markets and awareness using the 5Ps and the “priority assistive products” list as entry points (1, 6). This approach involves working nationally to demonstrate the value of AT and build capacity by stimulating demand by improving affordability and mobilising financing. Beyond expanding access to AT, the stimulated demand can increase stability, reducing the perceptions of risk for both suppliers and purchasers, which also supports local manufacturers. However, some participants felt that they found it challenging to see the effects of market shaping at country-level because of the fluctuating national and local incentives within their day-to-day programme activities.

Limited understanding about AT is regarded as a key factor hindering demand generation activities. Knowledge and expertise about what AT is, and who needs it, remains an issue and there was still a need to build capacity and understanding of those procuring AT. For example, often orders for transport chairs (mainly used in hospitals) which are not designed for the user to self-mobilise, are received rather than for user-propelled wheelchairs, and seating cushions are consistently missed off procurement requests. Such a lack of knowledge can increase non-use and abandonment, leading to “wheelchair graveyards”, and which can perpetuate market failures.

One participant highlighted their market shaping approaches eloquently,

“improving access to AT is less about finding the perfect products, but more about, aligning the demand, the system, the markets, so that this AT just becomes a routine part of the public service delivery”

#### 3.3.2 Innovation

Innovation was a strong theme emerging from the interviews, but respondents' opinions about how it should be integrated into market shaping strategies varied. One participant argued that rather than being about product development, innovation was about scaling in low-income settings. However, another pointed out, for innovations to progress beyond small pilot projects, and to drive scale, demand generation is essential.

Innovation, especially in low-income settings, moves beyond devices to include innovations in assessment and service delivery. An approach that was suggested by a one participant was training parents to deliver basic speech therapy, effectively task shifting from the professional to those closest to the child. However, this model still requires qualified speech and language therapists to deliver initial training to the parents, and these professionals are in vanishingly small supply in many low-income settings. This idea is gaining traction, however, with Tanzania and Rwanda considering pilot schemes.

In one country, service delivery innovation has been supported by holding “boot camps”, which bring together medical and education assessors, along with the National Council on Disability to support disability registration. The team explored procurement options for hearing aids for a “boot camp” which targeted 100 children. Procuring locally in this African country, the hearing aids cost the equivalent of around $310 per device. The same product was available on the supply catalogue at a unit cost of $118 which is a considerable saving.

However, national logistics remain challenging. The product catalogue price cannot consider freight costs and handling fees, which can inflate the final cost substantially, depending on scale of purchase in each shipment. Depending on context, because shipping times can be lengthy, it may not be workable for some projects and for the “boot camp” mentioned here they ended up procuring locally because of these logistical factors. A potential innovation suggested by the team would be what they termed “regional catalogues” with pre-vetted suppliers and regional stocking hubs. This has the potential to promote manufacture across the region and may reduce procurement and shipping times substantially. This would necessitate a whole new regional tendering process, potentially taking years. A more practical approach might be strengthening logistics solutions for each region, alongside work to ease country level logistical constraints.

Digital AT is a relatively new area for the UNICEF. In one African country UNICEF is developing a “mobile as AT”-adjacent app. This app will provide caregivers and frontline workers with information and support them to guide basic assessments of a child with disabilities. This app is still in development, but the long-term aim is to provide a means to connect app users to AT providers. UNICEF is also currently exploring innovations in procuring digital AT in humanitarian settings. This innovation would shift from its usual manufacturer-based tendering to a more flexible wholesale model, which could overcome the logistical issues highlighted above. Long lead-in times are particularly problematic in humanitarian settings which often evolve extremely quickly.

Despite challenges, participants reported feeling a “dynamism” in AT innovation. One person sees a lot of “startups or social enterprises actually trying to tailor assistive technology a little bit more and also trying to develop products that seem to be more affordable as well [as] locally made”. Another participant emphasised that innovation should be person-centred, not device-led. For innovations to have the greatest impact they need integrating within systems and supported by governance frameworks, which we will turn to next.

#### 3.3.3 Governance and financing

The increasing focus on AT, and the increasing number of actors across the AT ecosystem, means that effective governance and financing structures need to be established. From this perspective, stewardship by a global procurement leader is welcome and helpful to avoid inefficiencies and overlaps in the system. All interviewees underscored the importance of fostering political will for AT for effective market shaping, which UNICEF is prioritising as part of ATscale. As one participant stated, “one of the challenges with AT is it's a subject of everyone, which happens to be a subject of no one sometimes”.

Precisely because UNICEF as a public partner sees itself as a “convenor” in markets, it has the chance to draw in strong private partnerships and work with the private sector to try to bridge the unmet-need gap. Involving the private sector was one of the financing options for AT discussed by Tay-Teo et al. (22) in the scoping review, and this resonated with some interviewees, who felt that by opening markets could promote the involvement smaller or more local businesses. Others suggested that while it was initially necessary to involve the private sector, to promote sustainability, over time financing should shift increasingly to government funding through social protection or social welfare mechanisms.

At national levels funding often remains limited, with one person pointing out that “for many countries ‘donation’ is the budget line”. This shows that in many contexts AT continues to be deprioritised in the face of other competing population needs, and the reality of indebtedness for many countries. Without sufficient budgetary allocation, a country's ability to access AT from the supply catalogue remains limited and they may have to rely on donations which can be of poor quality.

Some argue that more capacity support and resources at a country level are a pre-requisite for market shaping success; this could be achieved through donor support or debt forgiveness. Others suggested that governments creating a friendly financial environment, such as giving devices tax exempt status, or tax breaks for companies locally producing products would stimulate supply in-countries and would have knock-on implications for availability and affordability of AT.

One participant argued that unchecked private sector market shaping for AT can lead to big private sector players monopolising markets, as was the case with one of the publications in the review (38). Monopoly can limit competition and can actively reduce choice, thereby not giving suppliers sufficient incentives to reduce market prices of AT. This is an area where UNICEF can play a pivotal role as a steward, using its own market shaping approaches such as at-scale procurement and demand aggregation to rebalance markets and keep them competitive.

Some questioned the legitimacy of market shaping because is driven by global actors rather than local contexts with one participant emphasising that market shaping is not value-free, asking “who is shaping whose market and what is their purpose?” They further argued that market shaping strategies emanating from the “global North” risk perpetuating a postcolonial agenda, particularly in settings with significant power imbalances that allow international actors to dictate priorities and shape market dynamics.

This underscores the perceived normative nature of market shaping, and whose interests are being served and who is being marginalised. As a public sector procurer with an open global tender process, UNICEF is able to overcome some of the tensions that arise through the private “deal matching” market shaping approach offered by other actors, where partnerships are brokered without transparency. Participants suggest that these issues should be acknowledged and addressed if market shaping is to be both legitimate and equitable.

Financing for AT does not always end at the device itself, and ongoing costs can perpetuate inequalities and dependency. One person highlighted a lack of clarity on the maintenance of the products in the supply catalogue. Hearing aid batteries were mentioned by several participants who argued that the ongoing need for batteries can perpetuate dependency and precarity.

Despite the varied opinions about the most effective governance and financing strategies for market shaping, there was broad agreement that progress towards robust and equitable AT markets is being made, but requires patience, with one person describing progress as “a long slow burn”.

#### 3.3.4 Data gaps

The gaps in robust empirical data were less explicitly discussed in the interviews. Imperfect information was seen as a barrier to market shaping. Lack of information and low-awareness leads to weak demand and artificially low estimate of need, as discussed in Danemayer et al. (37). Such a lack of robust data on need was an impediment to government strategic planning and resource allocation. One participant highlighted the need to create a sense of urgency for donors and governments through cost-benefit analyses and social benefit impact assessments. However, they pointed to a lack of economists working on this especially in lower resourced contexts to consistently calculate these losses. This lack of data impacts the budgets available to procure AT from the supply catalogue, instead reinforcing the reliance on donations that are often unpredictable, inappropriate or of poor quality.

A lack of institutional memory is a challenge for effective provision of AT, particularly in situations with high turnovers. This is often the case in humanitarian settings, where one participant reported that people stay in-post for a maximum of should this be two years. These individuals come with specific role and task and have other priorities than AT. Further, the participant argued, in situations where guidance on AT already exists, people can get lost or feel that without technical knowledge it is too daunting. UNICEF preserves institutional memory around market shaping through having specialised teams which have stable mandates, as well as explicit strategic policies and codified processes, and holding long-term arrangements with suppliers.

## 4 Discussion

The present paper has drawn together recent literature on market shaping, as well as a series of interviews with people working around the UNICEF market shaping initiative on AT. The scoping review identified several key themes—system-level thinking; innovation; governance and financing; and data gaps—which formed an analytical framework for a UNICEF case study.

UNICEF occupies a unique position in the market shaping space. As a public sector actor, one of its mandates is to promote affordable and quality products for all. Its equity-driven approach to market shaping differs substantially from more “traditional” profit-driven models.

UNICEF's convening role enables it to coordinate a wide range of actors to work to strengthen markets and ensure that people can access affordable and quality AT. More evidence is needed to assess long-term and sociological impacts of including AT in the supply catalogue, particularly for more recently added products. Without this, it may be difficult to present compelling business cases for governments to invest in national AT markets. This includes digital assistive products, which UNICEF is currently exploring, but which is “conceptually messy” (34, 37), making the impact of market shaping even more difficult to measure.

As one of the world's largest procurers, UNICEF can drive systemic change in the practice of market shaping. The case study reveals meaningful progress as well as areas of untapped potential, such as a 96% reduction in the price of hearing aids in Iran. These gains show how targeted public sector investment can influence AT markets to improve access and affordability. However, as presented, these gains are uneven, and currently data are patchy, but as the inclusion of AT in the supply catalogue is still in its early days, it is likely that similarly substantial price reductions will emerge as more data become available.

Innovation in the AT space encompasses products governance, finance, data and standards. Equity should be a core foundational principle of innovation, prioritising underserved populations in markets that may be smaller or less developed. For-profit innovation may privilege areas of the market that are most easily entered or with the highest perceived returns, meaning that highly specialised products may remain overlooked. To address this, a mission-led approach could align innovation and market shaping across interconnected systems to promote equitable access to “life-saving and life enabling” AT for all (24).

Market shaping is not without criticism. Some argue that it prioritises the agendas of donors and implementing actor priorities, which are often situated within the neoliberal assumption of private sector primacy. As this review has shown, however, these actors are not value neutral, nor are their interests necessarily aligned with those of AT users (38).

Reframing market shaping as part of an “AT access mission” may address some of these weaknesses. Mission-led approaches call for a fundamental shift in the how AT is perceived, valued and invested in (5, 24, 34, 48), reconceptualising AT as essential infrastructure for wellbeing and participation. Market shaping then becomes one of several tools for shaping the AT landscape and advancing global agendas such as UHC and the SDGs (24). This, to some extent, is the basis on which UNICEF and its partners, ATscale and AT2030 have attempted to centre market shaping within a wider programme of AT access.

### 4.1 Limitations

The scoping review was conducted by a single researcher, which may have led to some bias in screening, extraction and interpretation. To avoid this as much as possible, search strategies and search terms were agreed in advance with all team members.

The evidence base showed limited heterogeneity of authors. Much of the literature on AT market shaping comes from a small and sometimes recurring set of researchers, which may have resulted in a relatively narrow range of perspectives. This limited authorship also points to the fact that market shaping for AT is still a critically under-researched area. Despite this, the studies included in the covered a good range of thematic areas and market shaping issues.

The interviews took place over the course of 2 years, meaning that some insights in earlier conversations may be less relevant to the currently climate. Additionally, the changing climate around global development restricted the actors for interview significantly. Care was taken in analysis to avoid out-of-date information and ensure relevance, and while the relatively small number of people interviewed and the early stages of availability of pricing data may limit generalisability, the present data provides a valuable case study.

## 5 Conclusions

This scoping review and UNICEF case study, reveals a field that is rapidly evolving field that is still grappling with fundamental conceptual and practical concerns. Market shaping for AT has already shown its initial transformative potential in areas such as the significant price-reduction in hearing aids in Rwanda and Iran, This highlights the central role that public sector actors such as UNICEF can play in convening stakeholders and guiding markets to improve AT affordability and availability. However, such gains are not yet universal, and this in part requires implementation and investment to be redoubled by all global partners.

Market shaping may not be considered a “magic bullet” but global thinking about how this can apply to complex challenges is developing. There is growing recognition about the need to move beyond a narrative which seeks to “fix” broken markets and instead moves towards collective mission setting. Mazzucato (49) makes a strong case that mission-setting can be guided by a compass towards the common good. This includes collaboration, co-development of interventions, and sharing rewards. This approach of intentional proactive action is in line with UNICEF's strategy. This kind of agenda necessarily rises above single actors or approaches, to a collaborative assessment of the impact AT provision has on the wider goal of equity and social justice. Further research could deepen the mapping of AT to this approach as further data become available.

An “AT access mission” is an ambitious undertaking, but its promise for transformative change is significant. UNICEF's new strategy and its two-fold role as market steward and stakeholder convenor, has shown the potential to catalyse the coordinated and decisive action that is needed to achieve the ambition of getting quality, affordable AT to everyone who needs it. But no one partner can do this alone, and all actors will need to redouble efforts collaboratively and creatively, to ensure the lifechanging potential of AT can be realised in these challenging times.

## Data Availability

The datasets presented in this article are not readily available because anonymised data are not available. Requests to access the datasets should be directed to ellie.cole@ucl.ac.uk.
