# Supplementary material for: Market shaping for AT: scoping review and case study of an intervention
Source: Front Rehabil Sci. 2026 Sep 4;7:1918680. doi: 10.3389/fresc.2026.1918680 (PMC13587034; doi:10.3389/fresc.2026.1918680)
Supplement: Supplementary file 1 [file Supplementaryfile1.docx]

# Supplementary file one: PRISMA-ScR checklist

| **Item** | **Step** | **Checklist item** |
| --- | --- | --- |
| 1 | Title | Identify the report as a scoping review |
| 2 | Abstract | Provide a structured summary that includes (as applicable): background, objectives, eligibility criteria, sources of evidence, charting methods, results, and conclusions that relate to the review questions and objectives |
|  | **Introduction** | |
| 3 | Rationale | Describe the rationale for the review in the context of what is already known. Explain why the review questions/objectives lend themselves to a scoping review approach. |
| 4 | Objectives | Provide an explicit statement of the questions and objectives being addressed with reference to their key elements (e.g., population or participants, concepts, and context) or other relevant key elements used to conceptualise the review questions and/or objectives. |
|  | **Methods** | |
| 5 | Protocol and registration | Indicate whether a review protocol exists; state if and where it can be accessed (e.g., a Web address); and if available, provide registration information, including the registration number |
| 6 | Eligibility criteria | Specify characteristics of the sources of evidence used as eligibility criteria (e.g., years considered, language, and publication status), and provide a rationale. |
| 7 | Information sources | Describe all information sources in the search (e.g., databases with dates of coverage and contact with authors to identify additional sources), as well as the date the most recent search was executed |
| 8 | Search | Present the full electronic search strategy for at least one database, including any limits used, such that it could be repeated |
| 9 | Selection of sources | State the process for selecting sources of evidence (i.e., screening and eligibility) included in the scoping review. |
| 10 | Data charting | Describe the methods of charting data from the included sources of evidence (e.g., calibrated forms or forms that have been tested by the team before their use, and whether data charting was done independently or in duplicate) and any processes for obtaining and confirming data from investigators |
| 11 | Data items | List and define all variables for which data were sought and any assumptions and simplifications made. |
| 12 | Critical appraisal of sources of evidence | If done, provide a rationale for conducting a critical appraisal of included sources of evidence; describe the methods used and how this information was used in any data synthesis (if appropriate) |
| 13 | Synthesis of results | Describe the methods of handling and summarising the data that were charted |
|  | **Results** | |
| 14 | Selection of sources | Give numbers of sources of evidence screened, assessed for eligibility, and included in the review, with reasons for exclusions at each stage, ideally using a flow diagram |
| 15 | Characteristics of sources | For each source of evidence, present characteristics for which data were charted and provide the citation |
| 16 | Critical appraisal within sources | If relevant, report data for each source using the approach set out in step 12 |
| 17 | Results of each source | For each included source of evidence, present the relevant data that were charted that relate to the review questions and objectives |
| 18 | Synthesis of results | Summarise and/or present the charting results as they relate to the review questions and objectives. |
|  | **Discussion** | |
| 19 | Summary of evidence | Summarise the main results (including an overview of concepts, themes, and types of evidence available), link to the review questions and objectives, and consider the relevance to key groups |
| 20 | Limitations | Discuss the limitations of the scoping review process. |
| 21 | Conclusion | Provide a general interpretation of the results with respect to the review questions and objectives, as well as potential implications and/or next step |
| 22 | Funding | Describe sources of funding for the included sources of evidence, as well as sources of funding for the scoping review. Describe the role of the funders of the scoping review |
