# Supplementary material for: Market shaping for AT: scoping review and case study of an intervention
Source: Front Rehabil Sci. 2026 Sep 4;7:1918680. doi: 10.3389/fresc.2026.1918680 (PMC13587034; doi:10.3389/fresc.2026.1918680)
Supplement: Supplementary file 2 [file Supplementaryfile2.docx]

# Supplementary file two: database search strategy and search string

### Search strategy example

- Database: PubMed
- Date searched: 3rd April 2026
- Search fields: title and abstract
- Filters applied: English, journal publications, date (1st January 2018-3rd April 2026)

**Purpose:** to identify literature on AT and market shaping, building on the MacLachlan et al (2018) scoping review

**Search development:** initial search terms drafted by the first author and refined through iterative testing and review within the research team

### Search string

(("market shap*"[Title/Abstract] OR "market mak*"[Title/Abstract] OR "market suppl*"[Title/Abstract] OR "market dev*"[Title/Abstract] OR "market structur*"[Title/Abstract] OR "market stim*"[Title/Abstract] OR "market demand*"[Title/Abstract])

AND

("assistive"[Title/Abstract] AND ("tech*"[Title/Abstract] OR "prod*"[Title/Abstract] OR "equip*"[Title/Abstract] OR "device"[Title/Abstract]))) AND (y_10[Filter])
